# A Comparison of Functional Features of Chinese and US Mobile Apps for Pregnancy and Postnatal Care: A Systematic App Store Search and Content Analysis

**DOI:** 10.3389/fpubh.2022.826896

**Published:** 2022-02-17

**Authors:** Hongli Yu, Juan He, Xinghao Wang, Weilin Yang, Bo Sun, Anna Szumilewicz

**Affiliations:** ^1^Department of Sport, Gdańsk University of Physical Education and Sport, Gdańsk, Poland; ^2^Jiuling Primary School, Mianyang, China

**Keywords:** pregnancy, postnatal care, mobile app, China, functionalities, United State of America

## Abstract

**Background:**

Pregnancy to postpartum (PtP) applications (apps) are becoming more common tools to document everything from pregnancy and delivery to nutrient allocation, life taboos, and infant medical examinations. However, the dependability, quality, and efficacy of these apps remain unclear. This study examined the features and functions of mobile PtP care apps accessible in China and the United States and to identify the major gaps that need to be addressed.

**Methods:**

Apps were selected by searching the Apple App Store and Android Markets (in the US and China) for the terms “pregnancy” and “postpartum” in Chinese and English. The apps' security, quality, and effectiveness were investigated, and chi-square tests and analysis of variance were performed to examine the differences in characteristics between apps available in the US and China.

**Results:**

A total of 84 mobile PtP care apps (45 from the US and 39 from China) were included. A total of 89.7% (35/39) of Chinese mobile apps did not provide safety statements or supporting evidence. The objective app quality ratings for Chinese and US apps were 3.20 ± 0.48 (mean ± standard deviation) and 3.56 ± 0.45, respectively (*p* > 0.05). A greater number of Chinese apps provided app-based monitoring functions, namely recording fetal size (*n* = 18, 46.2% in China vs. *n* = 3, 6.7% in the US), contractions (*n* = 11, 28.2% in China vs. *n* = 0, 0% in the US), pregnancy weight (*n* = 11, 28.2% in China vs. 0, 0% in the US), and pregnancy check-up reminders (*n* = 10, 25.6% in China vs. *n* = 0, 0% in the US). Meanwhile, a greater number of US apps provided exercise modules, namely pregnancy yoga (*n* = 2, 5.1% in China vs. *n* = 21, 46.7% in the US), pregnancy workouts (n = 2, 5.1% in China vs. n = 13, 28.9% in the US), and pregnancy meditation (*n* = 0, 0% in China vs. 10, 22.2% in the US) (*p* < 0.01). A medium security risk was identified for 40% (18/45) of apps in the US and 82.1% (32/39) of apps in China (*p* < 0.01).

**Conclusions:**

The functionality and characteristics of in-store mobile apps for PtP care varied between China and the US. Both countries' apps, particularly Chinese apps, encountered issues related to a lack of evidence-based information, acceptable content risk, and program evaluations. Both countries' apps lacked proper mental health care functions. The findings suggest that the design of app features should be enhanced in both countries, and increased interaction between app creators and users is recommended.

## Introduction

The pregnancy to postpartum (PtP) stages are critical because, during this period, a woman's health is very vulnerable (1, 2). The 22nd Annual Meeting of the International Federation of Obstetrics and Gynecology discussed the present condition of PtP health care throughout the world. Experts noted that health care during the PtP period is imperative and encompasses a range of health care measures for the mother and fetus before, during, and after pregnancy, as well as during the puerperium (lactation) and neonatal phases (3).

During the PtP period, everything from pregnancy and delivery to nutrient allocation (4), life taboos (5), and newborn physical examinations (6) must be closely monitored. Pregnancy and postnatal education and support provided by a multidisciplinary team of specialists (e.g., doctors, midwives, trainers, and psychotherapists) may enhance the quality of care (7, 8). However, they may be too expensive or unavailable in impoverished countries (9, 10). Additionally, coronavirus disease 2019 (COVID-19) and its corresponding isolation periods may harm the health of pregnant and postpartum women (11). Thus, a lack of professional coaches, financial support, and infrastructure may hinder the provision of PtP care from a multidisciplinary team of specialists.

Technological advancements have facilitated the rapid rise of electronic health (eHealth) and mobile health (mHealth) in recent years (12, 13). According to a recent survey in Switzerland, PtP applications (apps) are becoming increasingly popular (14): 91% of parents use digital media to learn about their child's health and development (15). Evidence from randomized trials and comprehensive reviews indicates that mobile apps are typically beneficial for improving maternal physical health (e.g., weight management, mental health, and pregnancy awareness) (16–18), and affordability is a major advantage of such apps (19). However, the programs available in app stores are highly varied in terms of function, design, and overall quality, and they are not always subjected to rigorous evaluation through effective randomized controlled trials (RCTs) (18, 19). Meanwhile, systematic reviews of RCTs on maternal apps described the information for software and hardware, intervention content and delivery, and limitations. They still highlighted the risks of RTCs, such as unclear allocation concealment, no evaluation of objective quality in intervention apps, and no published protocols (20–22). Furthermore, studies reviewing apps that cover children's first 1,000 days of life (from conception to the age of 24 months) have exclusively focused on the prenatal or postnatal phases, ignoring the continuity between the two periods and their combined effect on the health of the mother and child (23, 24). For instance, taking notes of medical treatment received by both mother and infant, communicating with health professionals, as well as monitoring the mother's sleep and mental health are all uncommon features in apps (25). The dependability, quality, and efficacy of currently available PtP care apps are unclear, which may be a barrier to health promotion because pregnant and postpartum women are more susceptible to external influences (e.g., media coverage, apps information, social variables) (26). Incorrect information on health care and lifestyle may lead to unnecessary worry or stress during the perinatal period (26, 27).

Unsurprisingly, however, data supporting the effectiveness of mobile apps (26) are lacking because of the wide range of properties, responsibility for information correctness, degree of trustworthiness, and accessibility of content updates, as well as the absence of a certification mechanism or their categorization as a medical device (28). Furthermore, conventional app design and assessment procedures ignore the health literacy level of the target demographic and are disconnected from users' actual requirements (29, 30). Users' decisions may be influenced by variables such as an app's popularity, aesthetics, functionality, and user engagement (29, 30). However, one study revealed that many users did not critically evaluate the authenticity of the content offered by apps or consider problems related to their personal information and data (31).

Based on a review of recent studies of the impact of mHealth on the PtP period, two results emerged: (1) the importance of mothers receiving accurate health information throughout their children's first 1,000 days of life (2, 17) the major influence of mHealth on maternal well-being, lifestyle, and decision-making about pregnancy and infant health (32). Given these considerations, as well as the substantial gaps in the current research, several factors are unknown: (1) the authenticity, quality, and effectiveness of the most recent upgraded content provided by PtP care apps; (2) whether the apps consider privacy and security issues when collecting personal information and data. Therefore, a comprehensive study of current PtP care apps is timely and necessary during the COVID-19 pandemic.

Thus, the purposes of this study were to (1) describe and analyze the features and functions of mobile apps for PtP care available in China and the US, two of the largest app markets; (2) examine the apps' security, quality, and effectiveness; and (3) provide suggestions for future development and usage of mobile apps for PtP care of mothers and children throughout the first 1,000 days of life. On the basis of these goals, we anticipated that (1) the functions and features of PtP care apps in the US and China would be similar; (2) the latest upgraded content provided by apps would be high-quality and effective; and (3) all the apps would be concerned with the privacy and security of users' personal information and data.

## Materials and Methods

### Data Source

An electronic search of apps was performed from June 11 to August 5, 2021, using the keywords “pregnant woman, 9/9 months of pregnancy,” “birth,” “infant,” “baby,” “obstetrics,” “pregnancy,” “postpartum,” “new baby,” and “kid” in both English and Chinese languages. We selected English- or Chinese-language apps from the Apple App store (in China and the US), Google Android Play (in the US), Huawei Android Market (Huawei Holdings Limited, in China), Baidu Android Market (Baidu, Inc., in China), and 360 Android Market (Qihoo 360 Technology Co., Ltd, in China).

### App Selection Criteria and Data Extraction

The mobile apps for PtP care included in this study were defined as those that fulfilled any PtP health care needs. This study only included mobile apps with a minimum of 1 million downloads in the initial search list. Users are less likely to choose and download mobile apps with download numbers below this figure because they tend to choose the top mobile apps, which are ranked according to user comments and download count (28). The exclusion criteria were (1) duplicate and irrelevant apps (apps with the same name and producer were defined as duplicates, regardless of the availability of different versions); (2) apps without any meaningful introduction or instruction in the app store; (3) apps without a Chinese or English interface; (3) apps without a rating; (4) apps with fewer than 1 million downloads; (5) paid apps without trial; and (6) apps with no updates since January 1, 2020.

Two investigators independently chose the apps for inclusion in the study based on the inclusion criteria. The investigators extracted the following data from each included app: the app name, developer, specifications (medical, health, fitness, or unavailable), acquisition cost (free or in-app purchase), most recent update date, target users [women trying to conceive (TTC), pregnant women, postpartum women, those providing infant care, all types, or unspecified], safety statement (potential risks or “use under guidance” disclaimer), operating system (iOS or Android), supporting evidence (descriptive study, observational study, or randomized controlled trial), number of languages offered, user rating, and source of information (clinical guidelines). To avoid data omission, we compiled the aforementioned data into a spreadsheet, and each researcher used the same spreadsheet to document those data. Conflicts regarding inclusion and data extraction between the researchers were resolved through discussion.

### In-depth Analysis of Perinatal Care Apps

To investigate the selected apps in depth, the apps were downloaded and installed on iOS and Android devices. Six independent researchers were each assigned the same number of apps. Two independent researchers registered and logged into each app to evaluate the quality of its content and functions; this ensured that each program was reviewed on both Apple App Store and the Android markets (Google, Huawei, Baidu, and 360). Where relevant, simulated input data, such as the predicted birth date and the start of the previous menstrual cycle, were utilized to accurately analyze the app's potential. For a comprehensive assessment of the functions of apps over the full pregnancy duration, two researchers pretended to be in the first trimester of pregnancy, two in the second trimester, and two in the third trimester. All investigators assessed and analyzed TTC and postpartum period information. The primary modules were recorded first, followed by the auxiliary functions inside each module after logging into the app. Each researcher utilized the same spreadsheet to document those data. Data on the apps' basic information of functionality and technological aspects were collected and analyzed between August 7 and October 20, 2021. The Fleiss Kappa value was used to measure the trustworthiness of the data gathered by the six researchers (33). The Mobile Application Rating Scale (MARS) was adopted to evaluate app quality through four dimensions of objective app quality, including engagement, functionality, aesthetics, and information quality (34). The subjective quality subscale of the MARS was omitted in the assessment since we aimed to assess the objective quality of apps. All MARS items were scored on a 5-point Likert scale ranging from 1 (inadequate) to 5 (excellent), with the researcher required to circle the number that most correctly indicated the quality of the app component under assessment (34). The mean scores for each dimension were calculated, and the mean total score of objective app quality was calculated across all 4 dimensions (34). Apps that scored ≥3 out of 5 on the MARS were considered acceptable quality, and scores higher than 4 were rated as high quality (35). Three independent MARS-trained reviewers scored ratings to the apps. Disparities and uncertainties regarding the scores of apps among the researchers were addressed through discussion and reached a consensus on the final MARS scores. The component, runtime, and communication security of all apps were examined by the national anti-fraud center (version 1.1.17), which was established by China's Ministry of Public Security in 2021 (36). Some of its fundamental capabilities are anti-fraud early warning, identification verification, app self-examination, and risk inquiry, which effectively check the security performance of App and reduce the possibility of individuals being swindled (36). Scores <60 out of 100 on the test report represented a high risk, scores ≥60 and ≤ 80 represented a medium risk, and scores >80 represented low risk (36).

### Statistics Analyses

The baseline features were summarized using Origin (2021) (Northampton, MA, USA), and a chi-square test or analysis of variance with a significance threshold of 0.05 was performed using OpenEpi (version 3.01) to compare the Chinese and US apps (37). The module's frequencies and percentages were calculated using Origin (2021) (Northampton, MA, USA). Further, a chi-square test with a significance threshold of 0.05 was performed using OpenEpi (version 3.01) to analyze the functional differences between the Chinese and US apps.

## Results

### Basic Characteristics of the Included Apps

**Figure 1:**
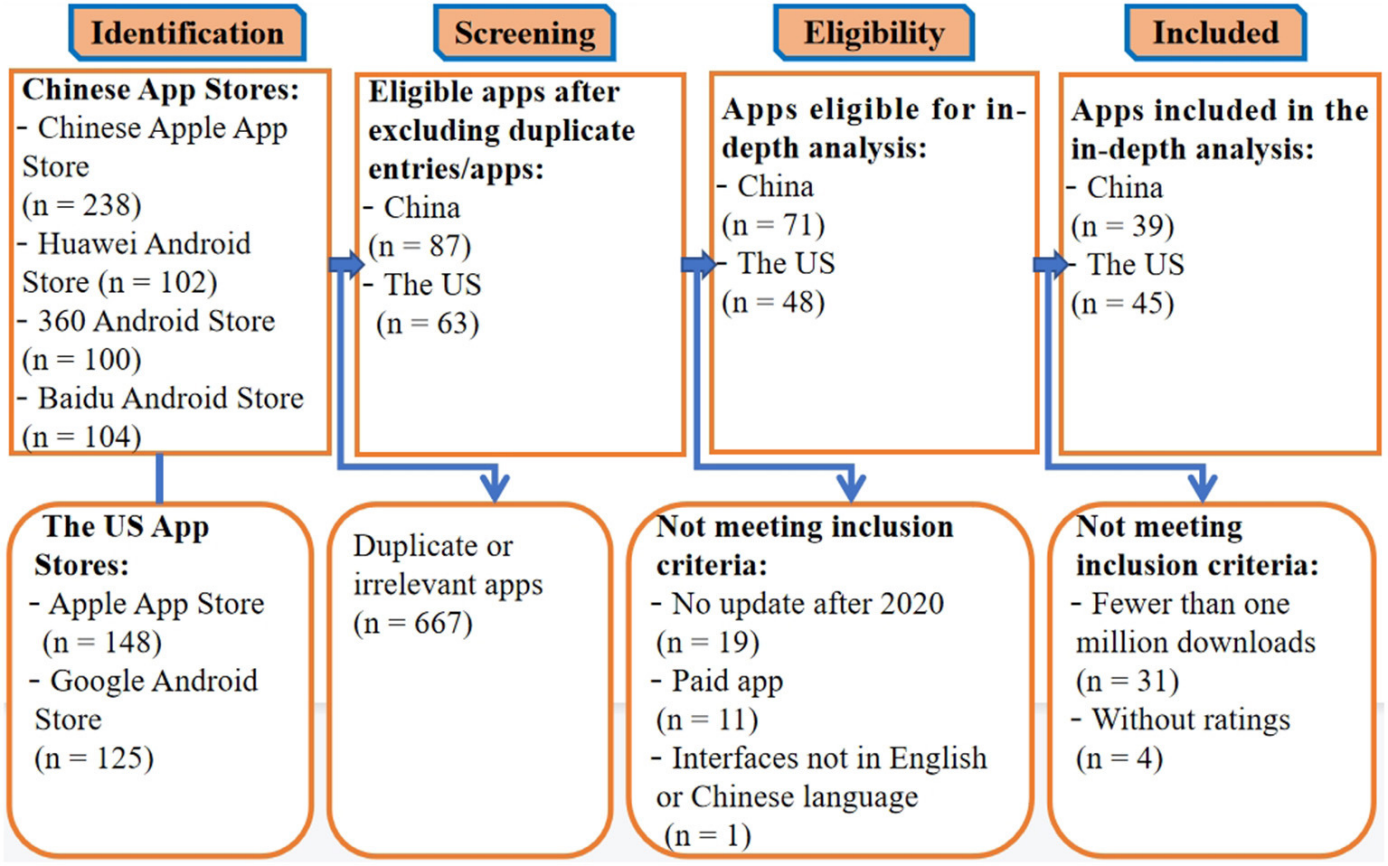
Flowchart of the app selection process.

Following a search of the Apple App Store, Google Play Store, Baidu Android Store, 360 Android Store, and Huawei Android Store, 817 apps were identified. After extensive screening, a total of 84 apps (39 from China and 45 from the US) were included. The flowchart in Figure 1 illustrates the app selection process.

The characteristics of all the included apps are summarized in Table 1. Among the Chinese mobile apps, 10.3% (4/39) were classified as medical, 79.4% (31/39) were classified as health and fitness, and 10.3% (4/39) were unclassified. A total of 89.7% (35/39) of Chinese mobile apps lacked clear safety statements and supporting evidence, whereas among the US mobile apps, 53.3% (24/45) provided clear safety declarations, and 51.1% (23/45) supplied supporting evidence (*p* < 0.01). In terms of acquisition costs, the Chinese app markets offered more free apps than did the US ones [66.7% (26/39) in China vs. 13.3% (6/45) in the US; *p* < 0.01]. Furthermore, a higher percentage of Chinese apps were targeted at women TTC and infant-care users compared with US apps [women TTC: 46.2% (18/39) in China vs. 13.3% (6/45) in the US; infant-care: 41% (16/39) in China vs. 2.2% (1/45) in the US; *p* < 0.05]. In addition, Android systems were more common in China than in the US [79.5% (31/39) in China vs. 31.1% (14/45) in the US; *p* < 0.01]. Multiple languages were available in 43.5% (17/39) of Chinese apps, whereas multiple languages were only available for only 20% (9/45) of apps in the US (*p* < 0.01). In the US, none of the apps was uncategorized; 86.7% (39/45) of mobile apps were labeled as “health and fitness”, whereas only 13.3% (6/45) were categorized as “medical”. The number of in-app updates did not differ between the US and China apps after the COVID-19 outbreak; in 2021, 62.2% (28/45) of US apps and 74.4% (29/39) of Chinese apps were updated. US apps had an average user rating of 3.41; this was slightly higher than that of the Chinese apps, which had a mean user rating of 3.09 (*p* > 0.05).

**Table 1:** Characteristics of the 84 apps for pregnancy to postpartum care identified in the US–China comparison.

**Category**	**China (*n* = 39)**	**United States (*n* = 45)**	**χ^2^/*F***	***p***
**Specifications**, ***n*** **(%)**				
**Medical**	**4 (10.3)**	**6 (13.3)**	**1.158**	***p* > 0.05^b^**
**Health and fitness**	**31 (79.4)**	**39 (86.7)**		
**NA^d^**	**4 (10.3)**	**0 (0)**		
**Acquisition costs**, ***n*** **(%)**				
**Free**	**26 (66.7)**	**6 (13.3)**	**25.200**	*p <* 0.01^**^^b^
**In-app purchase**	**13 (33.3)**	**39 (86.7)**		
**Target users (app description accompanying a clear statement)**, ***n*** **(%)**				
**Women TTC^a^**	**18 (46.2)**	**6 (13.3)**	**13.64**	*p* < 0.05^*^^b^
**Pregnant women**	**28 (71.8)**	**32 (71.1)**		
**Postpartum women**	**16 (41)**	**23 (51.1)**		
**Those providing infant care**	**16 (41)**	**1 (2.2)**		
**Not specified**	**3 (7.7)**	**2 (4.4)**		
**Safety statement**, ***n*** **(%)**				
**With**	**4 (10.3)**	**24 (53.3)**	**17.446**	*p* < 0.01^**^^b^
**Without**	**35 (89.7)**	**21 (46.7)**		
**Privacy policy**, ***n*** **(%)**				
**With**	**35 (89.7)**	**42 (93.3)**	**0.039**	***p* > 0.05^b^**
**Without**	**4 (10.3)**	**3 (6.7)**		
**Supporting evidence**, ***n*** **(%)**				
**With**	**4 (10.3)**	**23 (51.1)**	**15.988**	*p* < 0.01^**^^b^
**Without**	**35 (89.7)**	**22 (48.9)**		
**Operating system**, ***n*** **(%)**				
**iOS**	**29 (74.3)**	**33 (73.3)**	**26.820**	*p* < 0.01^**^^b^
**Android**	**31 (79.5)**	**14 (31.1)**		
**Year of the most recent update**, ***n*** **(%)**				
**2020**	**10 (25.6)**	**17 (37.8)**	**1.411**	***p* > 0.05^b^**
**2021**	**29 (74.4)**	**28 (62.2)**		
**Language**, ***n*** **(%)**				
**Chinese**	**22 (56.4)**	**0**	**63.562**	*p* < 0.01^**^^b^
**English**	**0**	**36 (80)**		
**Multiple languages offered**	**17 (43.6)**	**9 (20)**		
**Mean user rating (stars/5)**	**3.09 ± 0.70**	**3.41 ± 0.9**	**3.198**	***p* > 0.05^c^**

a *Trying to conceive.*

b *Chi-square test.*

c *Analysis of variance.*

d *Not available.*

** *Extremely significant difference at p < 0.01.*

* *Significant difference at p < 0.05*.

### Consistency of the Collected Content and Function Data of PtP Care Apps

Six independent researchers were assigned the same number of apps and were tasked with collecting the apps' information of functionality and technical characteristics. The Fleiss Kappa value was 0.8403, indicating that the authenticity and reliability of the apps' collected information of functions and technical characteristics were acceptable and could be analyzed.

### Functions and Modules

**Figure 2:**
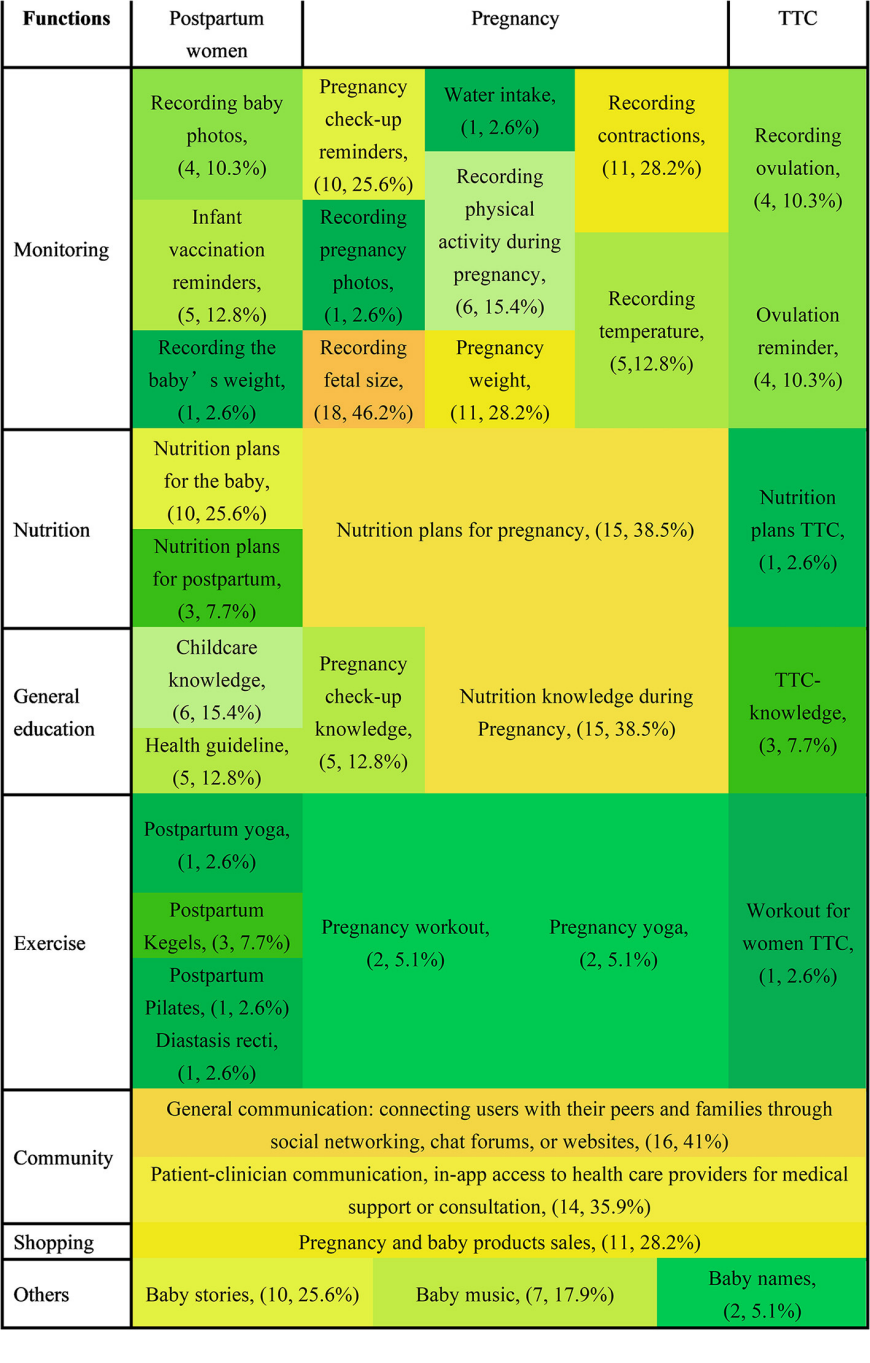
Heat map of features of the 39 Chinese mobile apps for pregnancy to postnatal care. TTC, trying to conceive.

**Figure 3:**
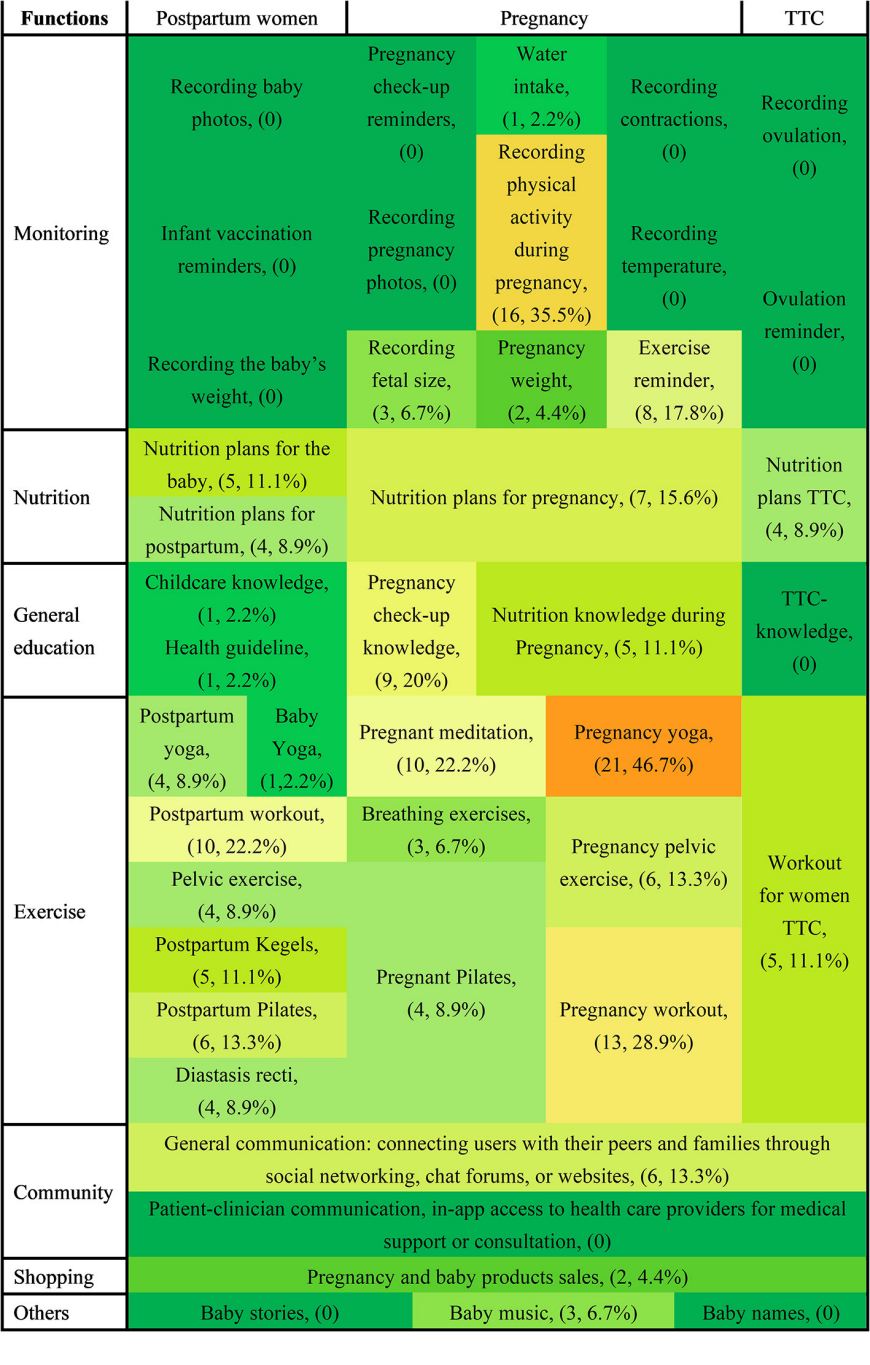
Heat map of features of the 45 US mobile apps for pregnancy and postnatal care. TTC, trying to conceive.

The features of each function provided by the Chinese and US apps are represented in heat maps in Figures 2, 3, and the data are summarized in Table 2. Chinese and US PtP care mobile apps contained seven functions [monitoring (e.g., recording fetal size, tracking physical activity during pregnancy, water intake, recording baby photos), nutrition, general education, exercise, community, purchasing, and others] and were targeted at four applicable groups (women TTC, pregnant women, postpartum women, and those providing infant care). Fewer app functionalities were comparable between the two countries. The most common function of the 39 Chinese apps was fetal size monitoring (*n* = 18, 46.2%), followed by general communication (*n* = 16, 41%), nutrition planning for pregnancy (*n* = 15, 38.5%), and nutrition knowledge throughout pregnancy (15, 38.5%). The most common function of the 45 US mobile apps was pregnancy yoga (*n* = 21, 46.7%), followed by monitoring physical activity in pregnancy (*n* = 16, 35.5%) and exercise during pregnancy (*n* = 13, 28.9%). Furthermore, none of the 39 Chinese mobile apps provided functions related to baby yoga, postpartum Pilates, meditation, treatment for psychosocial concerns, breathing exercises, postpartum pelvic exercises, pregnancy Pilates, or pelvic exercise. The following features were not included in any of the 45 US mobile apps: recording baby photos, pregnancy check-up reminders, recording contractions, recording ovulation date, infant vaccination reminders, recording pregnancy photos, recording temperature, ovulation reminders, recording the baby's weight, or addressing psychosocial concerns.

**Table 2:** Comparison of the characteristics of the mobile apps for pregnancy and postnatal care between the US and China.

**Category**	**China (*n* = 39)**	**United States (*n* = 45)**	**χ^2^**	***p***
**Log**, ***n*** **(%)**				
Recording baby photos	4 (10.3)	0 (0)		
Infant vaccination reminders	5 (12.8)	0 (0)	54.486	*p* < 0.01^**^
Recording the baby's weight	1 (2.6)	0 (0)		
Pregnancy check-up reminders	10 (25.6)	0 (0)		
Recording pregnancy photos	1 (2.6)	0 (0)		
Recording fetal size	18 (46.2)	3 (6.7)		
Water intake	1 (2.6)	1 (2.2)		
Recording physical activity during pregnancy	6 (15.4)	16 (35.5)		
Exercise reminder	0 (0)	8 (17.8)		
Pregnancy weight	11 (28.2)	2 (4.4)		
Recording contractions	11 (28.2)	0 (0)		
Recording temperature	5 (12.8)	0 (0)		
Recording ovulation	4 (10.3)	0 (0)		
Ovulation reminder	4 (10.3)	0 (0)		
**Nutrition**, ***n*** **(%)**				
Nutrition plans for baby	10 (25.6)	5 (11.1)		
Nutrition plans for postpartum women	3 (7.7)	4 (8.9)	25.200	*p* < 0.01^**^
Nutrition plans for pregnant women	15 (38.5)	7 (15.6)		
Nutrition plans for women TTC^a^	1 (2.6)	4 (8.9)		
**General education**, ***n*** **(%)**				
Childcare knowledge	6 (15.4)	1 (2.2)		
Health guideline	5 (12.8)	1 (2.2)	91.13	*p* < 0.01^**^
Pregnancy check-up knowledge	5 (12.8)	9 (20)		
Nutrition knowledge during pregnancy	15 (38.5)	5 (11.1)		
TTC knowledge	3 (7.7)	0 (0)		
**Exercise classes**, ***n*** **(%)**				
Postpartum yoga	1 (2.6)	4 (8.9)		
Postpartum workout	0 (0)	10 (22.2)	95.356	*p* < 0.01^**^
Pelvic exercise	0 (0)	4 (8.9)		
Postpartum Kegels	3 (7.7)	5 (11.1)		
Postpartum Pilates	1 (2.6)	6 (13.3)		
Diastasis recti	1 (2.6)	4 (8.9)		
Pregnancy meditation	0 (0)	10 (22.2)		
Pregnancy yoga	2 (5.1)	21 (46.7)		
Pregnancy pelvic exercise	0 (0)	6 (13.3)		
Pregnancy workout	2 (5.1)	13 (28.9)		
Pregnancy Pilates	0 (0)	4 (8.9)		
Workout for women TTC	1 (2.6)	5 (11.1)		
**Community**, ***n*** **(%)**				
General communication	16 (41)	6 (13.3)		
Patient–clinician communication	14 (35.9)	0 (0)	8.288	*p* < 0.01^**^
**Shopping**, ***n*** **(%)**				
With shopping function or service	11 (28.2)	2 (4.4)	9.017	*p* < 0.01^**^
**Others**, ***n*** **(%)**				
Baby stories	10 (25.6)	0 (0)		
Baby music	7 (17.9)	3 (6.7)	35.122	*p* < 0.01^**^
Baby names	2 (5.1)	0 (0)		

a *Trying to conceive.*

** *Extremely significant difference at p < 0.01*.

Our quantitative analysis revealed the following significant differences between China and the US in terms of the functions offered by the PtP care apps. (1) Monitoring: recording fetal size (*n* = 18, 46.2% in China vs. *n* = 3, 6.7% in the US) and tracking physical activity during pregnancy (*n* = 6, 15.4% in China vs. *n* = 16, 35.5% in the US); (2) nutrition: nutrition planning during pregnancy (*n* = 15, 38.5% in China vs. *n* = 7, 15.6% in the US); (3) general education: nutrition knowledge during pregnancy (*n* = 15, 38.5% in the China vs. *n* = 5, 11.1% in the US); (4) exercise: pregnancy yoga (*n* = 2, 5.1% in China vs. *n* = 21, 46.7% in the US), pregnancy workouts (*n* = 2, 5.1% in China vs. *n* = 13, 28.9% in the US), and pregnancy meditation (*n* = 0, 0% in China vs. *n* = 10, 22.2% in the US); (5) community: general communication (*n* = 16, 41% in China vs. *n* = 6, 13.3% in the US) and patient–clinician communication (*n* = 14, 35.9% in the China vs. *n* = 0, 0%); (6) shopping: pregnancy and baby product sales (*n* = 11, 28.2% in China vs. *n* = 2, 4.4% in the US), (7) others: baby stories (*n* = 10, 25.6% in the China vs. *n* = 0, 0% in the US) and baby music (*n* = 7, 17.9% in the China vs. *n* = 3, 6.7% in the US).

### App Quality Based on the MARS

The overall MARS scores for app quality ranged from a minimum score of 2.7 to a maximum of 4.4 (median 3.5) in the US vs. 2.3 to 4.2 (median 3.2) in China, with most apps (34/45, 76% in the US vs. 23/39, 59% in China) achieving a score >3. The engagement score ranged from 2.2 to 4.4 (median 3.6) in the US vs. 1.2 to 4.4 (median 2.9) in the China. The functionality score ranged from 3.0 to 4.8 (median 4.0) in the US vs. 2.5 to 4.5 (median 3.7) in the China. The esthetics score ranged from 2.3 to 4.4 (median 3.8) in the US vs. 2.3 to 4.7 (median 3.6) in the China, and the information score ranged from 1.2 to 4.9 (median 2.9) in the US vs. 1.2 to 4.2 (median 2.4) in the China. The mean MARS scores for all 84 analyzed Chinese and US apps are reported in Supplementary Table 1.

### Risk Assessment of PtP Care Apps in the US and China

The security risks associated with the 84 apps were assessed using the anti-fraud software. Among US mobile apps, 8.9 and 40% were considered high- and medium-risk, respectively; among Chinese apps, 7.6% (3/39) were deemed to be high-risk (Table 3). The risk of PtP care apps differed significantly between the US and China (*p* < 0.01; Table 3). According to the risk assessment report, “high-risk” generally suggests that the program includes hazardous information or third-party plug-ins, whereas “medium-risk” implies the possibility of personal information leakage.

**Table 3:** The risk assessment of pregnancy and postnatal care mobile apps in the US and China.

**Risk**	**United States (*n* = 45), *n* (%)**	**China (*n* = 39), *n* (%)**	***p*-value**
Low	23 (51.1)	4 (10.3)	*p* < 0.01^**^
Medium	18 (40)	32 (82.1)	*p* < 0.01^**^
High	4 (8.9)	3 (7.6)	*p* > 0.05

** *Extremely significant difference at p < 0.01*.

## Discussion

This study systematically demonstrated the features and functionalities of in-store mobile apps for PtP care in the US and China, two of the largest app marketplaces, offering an overview of their primary characteristics and functions with a special emphasis on weaknesses and gaps to be addressed with future eHealth-related innovations. We believe that such an investigation is essential for developing more effective PtP health apps during COVID-19. Pregnancy and postnatal education and support provided by a multidisciplinary team of specialists may improve the quality of care through apps, even among low-income groups or groups in impoverished areas. Overall, our study reveals that (1) there are differences in PtP care mobile apps between the two countries; (2) the quality and effectiveness of the information offered by apps must be carefully examined and combined with professional medical advice; and (3) some Chinese and US apps were suspected of stealing users' personal information. These findings do not support our hypothesis and highlight key areas for improvement of apps targeted at pregnant and postpartum women.

### Overview of Hypothesis Validation Findings

#### Metadata of Apps

All 45 US apps were classified as medical or health and fitness apps, but several Chinese apps (10.3%, 4/39) were uncategorized. This may be because the Food and Drug Administration (FDA) risk studies and policy recommendations for mobile health technology require apps to be categorized into three categories: health management, general management, and medical devices (38). Another possible explanation is that the FDA regulates all mobile health technology (38). Unlike the US, China lacks any such legislation. Additionally, few apps were classified as medical devices, indicating a severe weakness in Chinese–US apps. To overcome this issue, apps must not only be designed as information or entertainment tools but also be subjected to new medical device legislation. This legislation emphasizes the importance of anticipating the use of medical device apps by mothers and the role of these apps in controlling or assisting conception (39, 40). Furthermore, each Chinese app covered a broader range of target users than US apps. Three Chinese apps and two US apps did not specify their target users. By contrast, a few more US apps noted that it was vital for app developers to differentiate their products to match the specific demands of diverse populations (41). A previous study reported that pregnant and postpartum women in China had considerably different demands than did women in the US (5), which may explain the difference in target users.

Regarding supporting evidence and safety statements, only 10.3% of the Chinese apps offered references to the information provided and recognized their scientific responsibility. The mean value of the information was lower than the acceptable level in both the US and China. Pregnant and postpartum women may be more vulnerable to inaccurate information (26). Given the potential of mHealth apps to promote maternal health and knowledge about pregnancy and children's health and development, apps capable of providing the most comprehensive, accurate, and trustworthy information on pregnancy and the postnatal period are urgently needed.

Regarding acquisition costs, ~80% of US apps offered in-app purchases; this was connected to the difference in the exercise functions, as American apps offered plentiful training courses for pregnancy and exercise-related postpartum recovery. Exercise during pregnancy is increasingly popular in the US (42, 43), which may explain the large disparity in acquisition costs between the two countries. The addition of the fitness element in US apps is a worthwhile endeavor, particularly amid the current COVID-19 outbreak. Effective online prenatal exercise classes are useful to both mothers and their unborn children (11). Noticeable distinctions were observed between Chinese and US apps in terms of the operating system and language. Chinese apps tend to offer multiple languages and favor Android operating systems compared to their US counterparts. This may be related to China's increasing mobile market share in domestic and foreign markets in recent years (44).

User feedback is a true reflection of users' requirements and genuine experiences using the app (45, 46). Objective app quality reflects the app's information, functionality, aesthetics, and engagement (47). Both countries' user and objective app quality ratings were at acceptable levels, with the US marginally outperforming China in these categories and the engagement score of quality being poor in China. The development of PtP apps may be improved if the apps are highly valued and used by the target audience (46). The abovementioned results did not support our hypothesis that the most updated content offered by the app would be high-quality and effective. This suggests that the features of apps available in both the US and China should be improved, and more interaction between app developers and users is recommended.

#### Functions and Modules

According to our data, monitoring, nutrition, general education, exercise, community, shopping, and other features were the most utilized functions in prenatal care mobile apps in both China and the US. However, psychological services were seldom observed in either country's apps, even though psychological support is strongly related to pregnant and postpartum women's mental health (48). The primary impediment to developing this function and module may be its complicated nature (49).

Exercise-related features (e.g., pregnancy yoga, fitness, and meditation) were more frequent in US-based apps than in Chinese-based apps. We have already discussed the reasons for the discrepancies in the exercise modules during the pregnancy and postpartum periods. Research has demonstrated that exercise is beneficial during pregnancy (50), but because of a lack of funds and expert advice, many pregnant women do not achieve the recommended amount of physical activity (51, 52). Therefore, we propose that the Chinese apps should be upgraded to incorporate exercise modules to effectively address the aforementioned issues because online courses are less expensive than courses offered at professional organizations. This ensures that even in impoverished regions and during the COVID-19 lockdown, users can access relevant exercise modules (53).

Monitoring functionalities (e.g., tracking fetal size, physical activity during pregnancy, and contractions) were more common in Chinese apps than in US apps. These services are based on built-in algorithms (generally predictive modeling utilizing acquired personal data and possibly sophisticated methods, such as artificial intelligence) and provide users with immediate and direct advice depending on their circumstances (54). These functionalities play a crucial role, as offline healthcare may be unavailable during the COVID-19 pandemic (55). However, monitoring modules should be constructed with care and prudence, and further attempts with alternative algorithms should be encouraged simultaneously. The study findings also revealed that none of the apps directly enabled mothers to schedule appointments for medical treatment, immunization, or physical examinations, highlighting the absence of a direct link between apps and appointment scheduling systems. We recommend that PtP care apps be connected to a wider regional or national public health care network. This substantial gap could be solved by developing more networked and institutional apps to improve booking processes by lowering the number of calls or other request types from patients and the professionals that manage them (56).

#### Risk Assessment

Risk assessment is primarily concerned with two factors: the content and the app program. The content risk evaluation was primarily based on information quality, security statements, and scientific evidence. According to the findings of the information quality, supporting scientific evidence, and safety statement inquiries, the content-related risk was mostly due to a lack of safety statements and published scientific literature or had positive outcomes in studies that are not RCTs. This issue was more prominent in Chinese apps than in US apps. The app program checks revealed that several of these apps required the user to provide personal information beyond the extent of their permissions. The app self-examination by the national anti-fraud center measured the security of the installation package and program usage. The report demonstrated that certain apps compelled users to provide permission, claimed too many privileges, and gathered personal information that was outside of their scope. Analysts noted that mobile apps have a strong competitive edge in the market; apps would be unlikely to work if users do not accept the privacy policies (57). The above information does not support our hypothesis that all apps would be concerned with users' personal information and data privacy and security. We propose that users carefully read the privacy statements and that government agencies improve internet surveillance.

#### Strengths and Limitations

Several benefits and drawbacks of the present study should be considered when evaluating the conclusions. To the best of our knowledge, this study is the first to evaluate the quality and risks associated with in-store PtP care apps in China and the US. Second, we acted as users at various PtP stages to download and utilize the apps, which may or may not have resulted in overlooking functional information about the mobile apps. Third, the consistency and dependability of the data gathered were rigorously evaluated before being used. Finally, apps available in two countries with distinct cultures, healthcare systems (58, 59), and economic standings were analyzed. This study also has several drawbacks. First, we identified apps at a single time point, which may have omitted longitudinal updates to app features. Second, given the nature of the characteristics under investigation based on the user ratings, subjectivity in assigning ratings cannot be ruled out. Third, we excluded paid apps, which may have omitted some available information. Finally, we did not include apps from other countries because of linguistic barriers. Future researchers interested in this topic may wish to investigate different time periods to increase the reliability of longitudinal comparisons on functionality and technological aspects in more countries. Additionally, we will further analyze the influence of PtP apps on PtP health care.

## Conclusions

In summary, the in-store mobile PtP care apps differed between China and the US regarding their functionality and characteristics. Both countries' apps, and particularly Chinese apps, have their share of issues; these include lack of evidence-based information, risk of inaccurate content, and lack of app evaluation. Monitoring-related information was more common in Chinese apps, whereas exercise-related content was more abundant in US apps. Both countries' apps also failed to provide adequate mental health services. These findings highlight the need for improving the features of PtP care apps available in both countries, and extensive interaction between app developers and users is suggested. Maintaining a suitable level of regulation is necessary to ensure the quality and functionality of in-store apps. Basic public health services for women's health may improve with the development of high-efficiency PtP apps.

## Data Availability Statement

The original contributions presented in the study are included in the article/Supplementary Material, further inquiries can be directed to the corresponding author.

## Author Contributions

HY: conceptualization, funding acquisition, software, validation, visualization, and writing—original draft. HY, JH, XW, WY, and BS: data curation and formal analysis. HY and JH: methodology. HY and AS: project administration, resources, supervision, writing—review, and editing. All authors revised and approved the manuscript.

## Funding

Gdansk University of Physical Education and Sport supported this research.

## Conflict of Interest

The authors declare that the research was conducted in the absence of any commercial or financial relationships that could be construed as a potential conflict of interest.

## Publisher's Note

All claims expressed in this article are solely those of the authors and do not necessarily represent those of their affiliated organizations, or those of the publisher, the editors and the reviewers. Any product that may be evaluated in this article, or claim that may be made by its manufacturer, is not guaranteed or endorsed by the publisher.
