# Supplementary material for: A Comparison of Functional Features of Chinese and US Mobile Apps for Pregnancy and Postnatal Care: A Systematic App Store Search and Content Analysis
Source: Front Public Health. 2022 Feb 17;10:826896. doi: 10.3389/fpubh.2022.826896 (PMC8891489; doi:10.3389/fpubh.2022.826896)
Supplement: Supplementary file 1 [file Table_1.DOC]

**Supplementary Table 1.** Mobile Application Rating Scale scores for the 84 evaluated apps.

| Apps | Country | MARS score dimension | | | | |
| --- | --- | --- | --- | --- | --- | --- |
|  |  | Engagement mean score | Functionality mean score | Aesthetics mean score | Information mean score | App quality mean score |
| Juna: Wellness for Motherhood | US | 3.6 | 4.0 | 4.3 | 3.2 | 3.8 |
| Prenatal Yoga | Down Dog | US | 3.4 | 4.5 | 3.7 | 3.0 | 3.7 |
| Baby 2 Body: Pregnancy Wellness | US | 4.0 | 4.8 | 4.7 | 3.0 | 4.1 |
| Prenatal & Postnatal Workout | US | 3.6 | 3.5 | 4.3 | 3.5 | 3.7 |
| Prenatal & Mom Wellness App | US | 2.8 | 3.8 | 4.0 | 3.6 | 3.6 |
| Move your bump | US | 4.2 | 4.3 | 4.3 | 4.2 | 4.3 |
| Pregnancy Workouts- Prenatal | US | 4.2 | 4.5 | 4.7 | 3.3 | 4.2 |
| Pregnancy Yoga & Prenatal | US | 4.0 | 4.8 | 4.0 | 3.7 | 4.2 |
| Studio Bloom | US | 3.8 | 4.3 | 3.3 | 4.2 | 3.9 |
| Postnatal Pilates | US | 4.0 | 3.3 | 3.7 | 2.7 | 3.4 |
| Kegel Trainer | US | 3.2 | 3.5 | 3.3 | 1.8 | 2.9 |
| Diastasis Recti Fix | US | 2.8 | 3.5 | 3.0 | 2.2 | 2.8 |
| Care Pregnant Mother | US | 3.4 | 4.0 | 2.3 | 1.8 | 2.8 |
| Birthpedia | US | 4.2 | 4.6 | 4.7 | 2.3 | 3.9 |
| Birth Class | US | 3.8 | 3.8 | 2.7 | 1.8 | 3.0 |
| LUNA Mother Co | US | 3.6 | 4.0 | 3.3 | 1.7 | 3.1 |
| Preggi Bellies | US | 3.6 | 4.0 | 4.3 | 2.5 | 3.6 |
| Moms Into Fitness | US | 2.8 | 3.5 | 3.7 | 1.8 | 2.9 |
| SqueezyCX for Pelvic Health | US | 2.4 | 4.3 | 3.7 | 1.2 | 2.9 |
| Ma Yoga | US | 3.8 | 3.3 | 4.3 | 2.3 | 3.4 |
| Exercises for New Moms | US | 3.2 | 4.5 | 3.7 | 2.8 | 3.5 |
| Postnatal workouts | US | 4.4 | 4.5 | 4.3 | 1.2 | 3.6 |
| Empowered Motherhood Program | US | 4.2 | 4.3 | 4.7 | 3.5 | 4.1 |
| Hypnobirthing Prenatal Guide | US | 3.8 | 4.6 | 4.3 | 3.3 | 4.0 |
| Joy ON Kehel | US | 2.8 | 3.5 | 3.0 | 2.3 | 2.9 |
| Yoga Mamas | US | 3.2 | 3.5 | 3.7 | 2.5 | 3.2 |
| Tone It Up: Workout & Fitness | US | 3.2 | 3.8 | 3.3 | 4.0 | 3.5 |
| Hypnobirthing · Mom | US | 3.4 | 3.5 | 4.7 | 3.8 | 3.8 |
| Powermum - Pregnancy Workouts | US | 4.0 | 4.3 | 4.3 | 3.3 | 3.9 |
| Pregnancy Food Tracker ~Fittur | US | 2.2 | 3.3 | 3.7 | 2.7 | 3.0 |
| Pregnancy Workouts | US | 3.4 | 4.0 | 3.3 | 3.2 | 3.4 |
| Pregnancy Diet & Food Guide | US | 3.8 | 3.5 | 4.3 | 3.5 | 3.7 |
| HERA: Pregnancy & Motherhood | US | 4.2 | 4.5 | 4.0 | 4.5 | 4.3 |
| Carry: Pregnancy Workouts | US | 4.0 | 4.3 | 4.0 | 3.8 | 4.0 |
| Prenatal Yoga Pregnancy | US | 3.6 | 3.5 | 3.7 | 3.2 | 3.5 |
| My pregnancy | US | 3.2 | 3.8 | 3.3 | 4.2 | 3.6 |
| Pregnancy Workouts Exercises | US | 3.4 | 3.0 | 2.3 | 3.0 | 2.9 |
| Keleya | US | 4.2 | 4.3 | 4.7 | 2.5 | 3.925 |
| Pregnancy Exercise Program | US | 3.2 | 3.5 | 2.7 | 1.7 | 2.7 |
| YogiBirth | US | 3.6 | 4.5 | 3.3 | 1.8 | 3.3 |
| Every Mother | US | 4.2 | 4.3 | 4.3 | 4.9 | 4.4 |
| Diastasis Recti Workouts | US | 4.0 | 3.5 | 2.3 | 2.0 | 2.9 |
| Pregnancy & Baby Tracker WTE | US | 2.8 | 4.0 | 4.0 | 2.5 | 3.3 |
| HiMommy | US | 4.2 | 4.3 | 3.7 | 4.2 | 4.1 |
| Ovia Pregnancy Tracker | US | 3.2 | 3.0 | 3.3 | 1.7 | 2.8 |
| Yun Fu Yu Jia | China | 3.2 | 4.5 | 3.3 | 3.5 | 3.6 |
| G Dong | China | 3.0 | 3.8 | 2.3 | 4 | 3.2 |
| Ding Xiang Ma Ma | China | 3.4 | 4.3 | 4.7 | 3.7 | 4.0 |
| Bao Bao Shu | China | 4.2 | 4.0 | 4.3 | 4.2 | 4.1 |
| Ma Ma Wang Yun Yu | China | 4.4 | 4.5 | 4.0 | 1.8 | 3.6 |
| Yun Qi Ying Yang | China | 3.4 | 3.5 | 3.7 | 3.7 | 3.5 |
| Huai Yun Guan Jia | China | 3.6 | 3.3 | 4.3 | 2.5 | 3.4 |
| Da Du Pi Zhu Shou | China | 3.2 | 3.8 | 3.3 | 1.2 | 2.8 |
| Tai Jiao Gu Shi | China | 3.8 | 4.3 | 4.3 | 1.3 | 3.4 |
| Yun Yu Jia | China | 2.8 | 3.8 | 3.7 | 2.8 | 3.2 |
| Gong Suo Ji Lu | China | 2.6 | 3.0 | 2.7 | 3.5 | 2.9 |
| Ma Ma Bang | China | 3.8 | 4.5 | 4.3 | 2.5 | 3.7 |
| Yun Fu Shi Pu | China | 3.2 | 3.8 | 3.7 | 2.3 | 3.2 |
| La Ma Bang | China | 4.2 | 4.0 | 4.3 | 1.8 | 3.5 |
| Ke Xue Zuo Yue Zi | China | 3.2 | 4.3 | 3.3 | 3.2 | 3.5 |
| Yue Zi Shi Pu | China | 3.4 | 3.5 | 3.7 | 2.3 | 3.2 |
| Ma Ma She Qu | China | 3.8 | 4.3 | 3.7 | 2.8 | 3.6 |
| Jing Qi Guan Li | China | 1.2 | 3.8 | 2.7 | 1.8 | 2.3 |
| Da Yi Ma | China | 1.8 | 3.3 | 2.3 | 2.5 | 2.4 |
| Yun Bao | China | 2.0 | 2.8 | 3.3 | 2.3 | 2.6 |
| Wei Tai Xin | China | 2.8 | 3.0 | 2.7 | 2 | 2.6 |
| Yun Qi Zhi Nan | China | 3.2 | 3.8 | 2.7 | 2.3 | 3.0 |
| Yun Fu Wu You | China | 3.4 | 3.5 | 3.3 | 3.8 | 3.5 |
| Huai Yun Zhu Shou | China | 2.8 | 4.0 | 3.7 | 3 | 3.3 |
| Yun Fu Bei Yun | China | 2.2 | 2.8 | 4.3 | 1.7 | 2.7 |
| Yu Jia | China | 2.6 | 2.5 | 3.3 | 2.3 | 2.6 |
| Mei You | China | 4.0 | 4.0 | 4.7 | 1.7 | 3.6 |
| Yu Xue Yuan | China | 3.8 | 3.5 | 4.7 | 2 | 3.5 |
| Gai Ge Er | China | 2.0 | 3.3 | 3.7 | 1.8 | 2.7 |
| Yun Fun Shi Pu | China | 2.6 | 3.8 | 2.8 | 1.2 | 2.6 |
| Yun Qi Ban Lv | China | 2.0 | 4.5 | 3.7 | 1.3 | 2.8 |
| Feng Kuang Zao Ren | China | 1.8 | 4.3 | 3.7 | 1.7 | 2.8 |
| Da Yi Ma Yue Jing Qi | China | 2.2 | 3.5 | 3.3 | 2.3 | 2.8 |
| Yu Jia (Ban Mi) | China | 2.6 | 4.3 | 2.3 | 2.8 | 3.0 |
| Yun Qi Ti Xing | China | 3.0 | 3.8 | 4.3 | 1.2 | 3.1 |
| Nv Sheng Ri Ji | China | 2.8 | 3.3 | 3.3 | 3.3 | 3.2 |
| Lan Ting Peng Di Ji Gai Ge Er | China | 2.4 | 3.8 | 3.7 | 1.7 | 2.9 |
| FemometerYue Jing Qi Zhu Shou | China | 1.6 | 3.0 | 4.3 | 1.2 | 2.5 |
| Nian Gao Ma Ma | China | 1.8 | 2.5 | 2.3 | 2.5 | 2.2 |
